# Supplementary material for: Efficient Prediction of Vitamin B Deficiencies via Machine-Learning Using Routine Blood Test Results in Patients With Intense Psychiatric Episode
Source: Front Psychiatry. 2020 Feb 20;10:1029. doi: 10.3389/fpsyt.2019.01029 (PMC7044238; doi:10.3389/fpsyt.2019.01029)
Supplement: Supplementary file 1 [file DataSheet_1.docx]

**Supplementary Table 1 (related to Table 1). Divided patient distribution data (n = 497)**

Training set (admission from September 2015 to December 2016, n = 373)

|  | ICD-10 code | | | | | | | | | |  | VitB_1_ [ng/mL] | | |  | VitB_12_ [ng/L] | | |  | Folate [μg/L] | | |
| --- | --- | --- | --- | --- | --- | --- | --- | --- | --- | --- | --- | --- | --- | --- | --- | --- | --- | --- | --- | --- | --- | --- |
|  | F0 | F1 | F2 | F3 | F4 | F5 | F6 | F7 | F8 | F9 |  | <20 | <28 | <30^*^ |  | <150 | <180^*^ | <200 |  | <3.0 | <4.0^*^ | <5.0 |
| N | 20 | 18 | 218 | 41 | 12 | 0 | 27 | 17 | 20 | 0 |  | 11 | 62 | 80 |  | 28 | 61 | 84 |  | 23 | 57 | 111 |
| % | 5.4 | 4.8 | 58.4 | 11.0 | 3.2 | 0 | 7.2 | 4.6 | 5.4 | 0 |  | 2.9 | 16.6 | 21.4 |  | 7.5 | 16.4 | 22.5 |  | 6.2 | 15.3 | 29.8 |

Validation set (admission from January 2017 to August 2017, n = 124)

|  | ICD-10 code | | | | | | | | | |  | VitB_1_ [ng/mL] | | |  | VitB_12_ [ng/L] | | |  | Folate [μg/L] | | |
| --- | --- | --- | --- | --- | --- | --- | --- | --- | --- | --- | --- | --- | --- | --- | --- | --- | --- | --- | --- | --- | --- | --- |
|  | F0 | F1 | F2 | F3 | F4 | F5 | F6 | F7 | F8 | F9 |  | <20 | <28 | <30^*^ |  | <150 | <180^*^ | <200 |  | <3.0 | <4.0^*^ | <5.0 |
| N | 8 | 3 | 82 | 17 | 4 | 0 | 2 | 3 | 4 | 1 |  | 4 | 19 | 32 |  | 9 | 19 | 23 |  | 6 | 15 | 23 |
| % | 6.5 | 2.4 | 66.1 | 13.7 | 3.2 | 0 | 1.6 | 2.4 | 3.2 | 0.8 |  | 3.2 | 15.3 | 25.8 |  | 7.3 | 15.3 | 18.5 |  | 4.8 | 12.1 | 18.5 |

For further information about this table, see **Table 1**.

**Supplementary Table 2 (related to Table 2). Divided data of vitamin B deficiencies in sub-groups**

Training set

|  | F0 | F1 | F2 | F3 | F4 | F6 | F7 | F8 | F9 |
| --- | --- | --- | --- | --- | --- | --- | --- | --- | --- |
| VitB_1_ [ng/mL] | 6 | 2 | 46 | 9 | 3 | 6 | 5 | 3 | 0 |
| VitB_12_ [ng/L] | 4 | 3 | 40 | 4 | 3 | 1 | 4 | 2 | 0 |
| Folate [μg/L] | 4 | 5 | 28 | 5 | 5 | 2 | 4 | 4 | 0 |

Validation set

|  | F0 | F1 | F2 | F3 | F4 | F6 | F7 | F8 | F9 |
| --- | --- | --- | --- | --- | --- | --- | --- | --- | --- |
| VitB_1_ [ng/mL] | 3 | 2 | 24 | 2 | 0 | 1 | 0 | 0 | 0 |
| VitB_12_ [ng/L] | 1 | 1 | 13 | 3 | 0 | 0 | 0 | 1 | 0 |
| Folate [μg/L] | 1 | 2 | 10 | 1 | 0 | 1 | 0 | 0 | 0 |

For further information about this table, see **Table 2.**

**Supplementary Table 3 (related to Table 3). Divided dataset of age, sex, and 29 parameters**

Training set (admission from September 2015 to December 2016, n = 373)

| Parameters | Units | Mean | SD |  | UN | mg/dL | 12.6 | 6.3 |
| --- | --- | --- | --- | --- | --- | --- | --- | --- |
| Age | years | 42.2 | 15.3 |  | Cre | mg/dL | 0.7 | 0.2 |
| Sex | Woman | n = 177 |  |  | T.bil | mg/dL | 0.6 | 0.5 |
| WBC | ×10^3^/µL | 8.0 | 2.8 |  | Na | mmol/L | 139 | 3 |
| Hb | g/dL | 13.7 | 1.7 |  | Cl | mmol/L | 105 | 5 |
| Hct | % | 40.2 | 4.5 |  | K | mmol/L | 3.7 | 0.4 |
| MCV | fL | 89.4 | 5.1 |  | cor.Ca | mg/dL | 9.1 | 0.6 |
| Plt | ×10^4^/µL | 24.8 | 6.3 |  | CK | IU/L | 507 | 1290 |
| RDW.CV | % | 13.6 | 1.3 |  | AST | IU/L | 31 | 37 |
| Neu | % | 69 | 12 |  | ALT | IU/L | 26 | 24 |
| Lym | % | 24 | 10 |  | LDH | IU/L | 236 | 92 |
| Mono | % | 6 | 2 |  | ALP | IU/L | 226 | 86 |
| Eo | % | 1 | 2 |  | γGTP | IU/L | 37 | 63 |
| Baso | % | 0 | 0 |  | Glu | mg/dL | 111 | 39 |
| TP | g/dL | 7.2 | 0.6 |  | CRP | mg/dL | 0.4 | 0.9 |
| Alb | g/dL | 4.4 | 0.4 |  | TSH | μIU/mL | 1.7 | 2.6 |

Validation set (admission from January 2017 to August 2017, n = 124)

| Parameters | Units | Mean | SD |  | UN | mg/dL | 13.6 | 7.8 |
| --- | --- | --- | --- | --- | --- | --- | --- | --- |
| Age | years | 42.3 | 15.7 |  | Cre | mg/dL | 0.7 | 0.2 |
| Sex | Woman | n = 51 |  |  | T.bil | mg/dL | 0.7 | 0.4 |
| WBC | ×10^3^/µL | 8.9 | 2.7 |  | Na | mmol/L | 139 | 3 |
| Hb | g/dL | 13.8 | 1.6 |  | Cl | mmol/L | 105 | 4 |
| Hct | % | 40.6 | 4.4 |  | K | mmol/L | 3.7 | 0.4 |
| MCV | fL | 87.6 | 9.8 |  | cor.Ca | mg/dL | 9.1 | 0.4 |
| Plt | ×10^4^/µL | 25.3 | 6.3 |  | CK | IU/L | 536 | 1032 |
| RDW.CV | % | 13.2 | 1.1 |  | AST | IU/L | 32 | 27 |
| Neu | % | 72 | 10 |  | ALT | IU/L | 29 | 24 |
| Lym | % | 21 | 9 |  | LDH | IU/L | 246 | 90 |
| Mono | % | 6 | 2 |  | ALP | IU/L | 216 | 63 |
| Eo | % | 1 | 2 |  | γGTP | IU/L | 40 | 62 |
| Baso | % | 0 | 0 |  | Glu | mg/dL | 112 | 41 |
| TP | g/dL | 7.3 | 0.5 |  | CRP | mg/dL | 0.4 | 0.7 |
| Alb | g/dL | 4.4 | 0.4 |  | TSH | μIU/mL | 1.6 | 1.5 |

For further information about this table, see **Table 3**.

Abbreviations: WBC, white blood cell count; Hb, hemoglobin; Hct, hematocrit; MCV, mean corpuscular volume; RDW.CV, red blood cell distribution width-coefficient variation; Plt, platelet; Neu, neutrocyte fraction; Lym, lymphocyte fraction; Mono, monocyte fraction; Eo, eosinocyte fraction; Baso, basocyte fraction; TP, total protein; Alb, albumin; UN, urea nitrogen; Cre, creatinine; T.bil, total bilirubin; Na, sodium; Cl, chloride; K, potassium; cor.Ca, corrected calcium; CK, creatine kinase; AST, aspartate transaminase; ALT, alanine transaminase; LDH, lactate dehydrogenase; ALP, alkaline phosphatase; γGTP, γ-glutamyltransferase; Glu, plasma glucose; CRP, C-reactive protein; TSH, thyroid-stimulating hormone.

**Supplementary Table 4 (related to Table 4). Summary of sensitivity, specificity, and accuracy for the training set**

|  | vitB_1_ | vitB_12_ | Folate |
| --- | --- | --- | --- |
| Sensitivity | 1.0 | 1.0 | 1.0 |
| Specificity | 1.0 | 1.0 | 1.0 |
| Accuracy | 1.0 [1.0 – 1.0] | 1.0 [1.0 – 1.0] | 1.0 [1.0 – 1.0] |

For further information about this table, see **Table 4**.

**Supplementary Table 5 (related to Table 4). Sensitivities and specificities at other operating points**

With high sensitivity

|  | vitB_1_ | vitB_12_ | Folate |
| --- | --- | --- | --- |
| Sensitivity | 0.875 | 0.737 | 0.867 |
| Specificity | 0.457 | 0.343 | 0.413 |

With high specificity

|  | vitB_1_ | vitB_12_ | Folate |
| --- | --- | --- | --- |
| Sensitivity | 0.375 | 0.211 | 0.333 |
| Specificity | 0.924 | 0.981 | 0.963 |

For further information about this table, see **Table 4** and **Fig. 2**

**Supplementary Table 6. Subgroup analyses**

AUC performance of the classifiers trained and evaluated using the data collected from only the F2 population data (top) and the other populations (middle). P-values are calculated using Delong’s test. Low AUC performance of vitB_1_ in F2 may suggest some heterogeneity, which requires further study.

|  | vitB_1_ | vitB_12_ | Folate |
| --- | --- | --- | --- |
| AUC (F2) | 0.625 | 0.561 | 0.692 |
| AUC (Others) | 0.879 | 0.569 | 0.668 |
| p-value | 0.014 | 0.96 | 0.87 |

**Supplementary Table 7.** **AUC with different cut-off values**

AUC performance of random forest classifiers when different cut-off values were used to define the deficiency. Asterisks show predefined cut-off values.

vitB_1_ [ng/mL]

|  | vitB_1_ < 20 | vitB_1_ < 28 | vitB_1_ < 30^*^ |
| --- | --- | --- | --- |
| AUC | 0.779 | 0.687 | 0.716 |

vitB_12_ [ng/L]

|  | vitB_12_ < 150 | vitB_12_ < 180^*^ | vitB_12_ < 200 |
| --- | --- | --- | --- |
| AUC | 0.640 | 0.599 | 0.570 |

Folate [μg/L]

|  | Folate < 3.0 | Folate < 4.0^*^ | Folate < 5.0 |
| --- | --- | --- | --- |
| AUC | 0.788 | 0.796 | 0.683 |
